# Silicon-photomultiplier-based PET/CT reduces the minimum detectable activity of iodine-124

**DOI:** 10.1038/s41598-021-95719-8

**Published:** 2021-09-01

**Authors:** David Kersting, Walter Jentzen, Pedro Fragoso Costa, Miriam Sraieb, Patrick Sandach, Lale Umutlu, Maurizio Conti, Fadi Zarrad, Christoph Rischpler, Wolfgang Peter Fendler, Ken Herrmann, Manuel Weber

**Affiliations:** 1 Department of Nuclear Medicine, University Hospital Essen, University of Duisburg-Essen, Hufelandstrasse 55, 45147 Essen, Germany; 2 Department of Diagnostic and Interventional Radiology and Neuroradiology, University Hospital Essen, University of Duisburg-Essen, Essen, Germany; 3 Siemens Medical Solutions USA, Inc., Knoxville, TN USA; 4 German Cancer Consortium (DKTK, Partner Site Essen), Essen, Germany

**Keywords:** Thyroid cancer, Molecular medicine

## Abstract

The radioiodine isotope pair ^124^I/^131^I is used in a theranostic approach for patient-specific treatment of differentiated thyroid cancer. Lesion detectability is notably higher for ^124^I PET (positron emission tomography) than for ^131^I gamma camera imaging but can be limited for small and low uptake lesions. The recently introduced silicon-photomultiplier-based (SiPM-based) PET/CT (computed tomography) systems outperform previous-generation systems in detector sensitivity, coincidence time resolution, and spatial resolution. Hence, SiPM-based PET/CT shows an improved detectability, particularly for small lesions. In this study, we compare the size-dependant minimum detectable ^124^I activity (MDA) between the SiPM-based Biograph Vision and the previous-generation Biograph mCT PET/CT systems and we attempt to predict the response to ^131^I radioiodine therapy of lesions additionally identified on the SiPM-based system. A tumour phantom mimicking challenging conditions (derived from published patient data) was used; i.e., 6 small spheres (diameter of 3.7–9.7 mm), 9 low activity concentrations (0.25–25 kBq/mL), and a very low signal-to-background ratio (20:1). List-mode emission data (single-bed position) were divided into frames of 4, 8, 16, and 30 min. Images were reconstructed with ordinary Poisson ordered-subsets expectation maximization (OSEM), additional time-of-flight (OSEM-TOF) or TOF and point spread function modelling (OSEM-TOF+PSF). The signal-to-noise ratio and the MDA were determined. Absorbed dose estimations were performed to assess possible treatment response to high-activity ^131^I radioiodine therapy. The signal-to-noise ratio and the MDA were improved from the mCT to the Vision, from OSEM to OSEM-TOF and from OSEM-TOF to OSEM-TOF+PSF reconstructed images, and from shorter to longer emission times. The overall mean MDA ratio of the Vision to the mCT was 0.52 ± 0.18. The absorbed dose estimations indicate that lesions ≥ 6.5 mm with expected response to radioiodine therapy would be detectable on both systems at 4-min emission time. Additional smaller lesions of therapeutic relevance could be detected when using a SiPM-based PET system at clinically reasonable emission times. This study demonstrates that additional lesions with predicted response to ^131^I radioiodine therapy can be detected. Further clinical evaluation is warranted to evaluate if negative ^124^I PET scans on a SiPM-based system can be sufficient to preclude patients from blind radioiodine therapy.

## Introduction

Radioiodine therapy has been a cornerstone of treatment of differentiated thyroid cancer (DTC) patients for decades. In both current American Thyroid Association (ATA)^1^ and European Association of Nuclear Medicine (EANM)^2^ guidelines for the management of DTC patients, the radioiodine therapy is not only recommended after initial thyroidectomy but also in case of recurring unresectable radioiodine-avid tumour lesions. Typically, radioiodine uptake is verified by planar or tomographic gamma camera imaging using low ^131^I activities in the range of 74–185 MBq^1^. However, in cases of rising thyroglobulin levels and negative radioiodine scans, therapy management becomes challenging. In these instances, “blind” radioiodine therapy is a controversially discussed option^1^. In a 2014 published survey among ATA members, 15% to 52% would perform radioiodine therapy, even if diagnostic ^123^I whole body scans are negative^3^. On the one hand, more than half of DTC patients show pathologic radioiodine uptake after blind therapy^3,4^, on the other hand, radioiodine therapy is not free of adverse effects, e.g., gastrointestinal symptoms, sialadenitis, secondary cancers, or bone marrow suppression^5^. These aspects show the demand for improved diagnostic approaches to identify patients, who might benefit from radioiodine therapy.

Whole-body ^124^I positron emission tomography (^124^I PET) after application of typical activities in the range of 25 to 74 MBq can be alternatively performed and its detectability was described as superior to diagnostic^6–8^ and comparable^9^ or superior^10^ to intra-therapeutic ^131^I gamma camera imaging. Moreover, this imaging modality allows for pre-therapeutic dosimetry as a theranostic approach^11,12^ to optimize the individual therapeutic activity.

However, reports of undetected lesions in ^124^I PET raised concern about the applicability of ^124^I PET to preclude patients from blind radioiodine treatment^13,14^. Undetected lesions may be explained by technical factors, e.g., a limited sensitivity of the PET/CT scanner^15,16^. Recently, technical improvements have become available in the field of PET detector technologies. New-generation silicon-photomultiplier (SiPM)-based “digital” PET systems outperform photomultiplier tube (PMT)-based “analog” PET systems in detector sensitivity and show improvements in detectability for various tracers, especially with regards to small and low-uptake lesions^17–21^.

The hardware technical improvements have enabled key reconstruction software advances, for example the highly improved, noise suppressing, TOF reconstruction with very narrow time kernels, which offers new opportunities for clinical PET^22^. Moreover, other advanced reconstruction methods have progressed in parallel, improving noise level, contrast recovery, detectability. In particular, it is worth mentioning: resolution recovery or point spread function (PSF) reconstruction, which recovers spatial resolution compensating for penetration effects in the scintillator crystals^23,24^; positron range correction, which improves spatial resolution and therefore detectability for tracers with high energy positrons^25–27^; maximum-a-posteriori (MAP) reconstruction, which allows to increase convergence and to contain the noise at the same time^28,29^.

We hypothesise that additional lesions (possibly not detectable in previous-generation PMT-based systems) that are treatable by radioiodine therapy can be identified in ^124^I PET by usage of a SiPM-based PET/CT system. Hence, we performed phantom measurements under challenging, but clinically realistic conditions, on both a SiPM- and a PMT-based system using a small tumour phantom with spherical inserts covering the typical size of small lymph node metastases and low activity concentrations.

Thus, the primary aim of this phantom study was to compare the size-dependant minimum detectable ^124^I activity (MDA) between the SiPM-based and the PMT-based PET/CT systems for different emission times and image reconstruction algorithms. In addition, we attempt to predict the response to radioiodine therapy of those lesions that are additionally identified on the SiPM-based system (at standard and at longer emission time durations).

## Methods

### PET/CT systems

Measurements were performed using two PET/CT scanners (Siemens Healthineers, Erlangen, Germany) with time-of-flight (TOF) modelling option: a new-generation Biograph Vision 600 and a previous-generation Biograph mCT, which are called Vision and mCT systems in this study, respectively. A short description of the scanner specifications is shown as Supplemental Material (Supplemental Table S1). In short, the Vision is equipped with SiPM detectors, a crystal size of 3.2 mm, time coincidence resolution of 214 ps, field of view of 26.3 cm, energy window of 435–585 keV, and NEMA sensitivity of 16.4 cps/kBq. In comparison, the mCT is equipped with PMT detectors, a crystal size of 4.0 mm, time coincidence resolution of 540 ps, field of view of 21.8 cm, energy window of 435–650 keV, and NEMA sensitivity of 9.7 cps/kBq.

### Small tumour phantom and its preparation

#### Small tumour phantom

**Figure 1:**
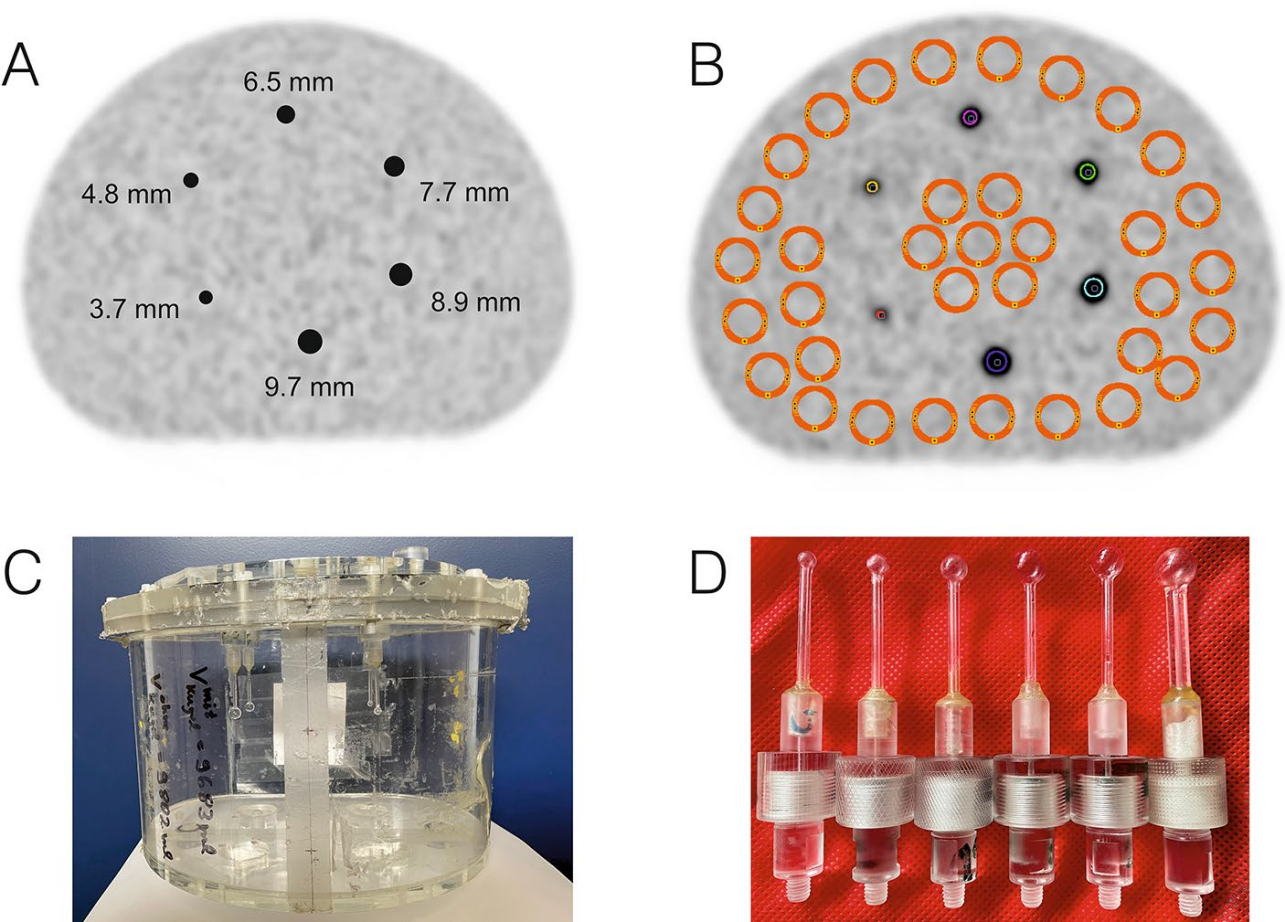
**(A)** Schematic representation of the small tumour phantom with small spherical inserts (sphere diameters are indicated). **(B)** Positions of the background ROIs (orange) and sphere VOIs (multiple colours). **(C)** Pictures of the small tumour phantom and **(D)** pictures of the spherical inserts.

The small tumour phantom consists of an abdominal torso NEMA phantom (Data Spectrum Corporation, Durham, USA) to simulate the patient body and of six refillable glass spheres to simulate small tumours (manufactured in our institution). Both a schematic representation of the small tumour phantom and pictures of the phantom and the small spherical inserts are shown in Fig. 1. The small spheres were mounted on a lid in a circle with a 6-cm radius. The centre of each sphere was positioned centrally within the phantom (approximately 10 cm from the lid). The inner diameters (inner volumes) were 3.7 mm (27 µl), 4.8 mm (58 µl), 6.5 mm (144 µl), 7.7 mm (239 µl), 8.9 mm (369 µl), and 9.7 mm (478 µl) and the inner cavity volume (with mounted spheres) was 9748 mL. The glass thickness of the spheres was 0.7 ± 0.2 mm.

Of note, the selected sphere diameters overlap well with the basic diameter range of 2–15 mm for normal lymph nodes in the head–neck region^30^. In addition, our group^31^ demonstrated that three quarters of the lymph nodes metastases observed in thyroid cancer patients were smaller than 11.5 mm (also in well agreement with findings in the basic literature, that is, that approximately 75% of normal lymph nodes in the head–neck region are smaller than 10 mm).

#### Phantom preparation

The spheres and the cavity of the phantom contained radioactive solution of ^124^I. The non-standard positron-emitter ^124^I was delivered by DSD pharma GmbH (Purkersdorf, Austria). The activity calibration measurements using a dose calibrator CRC-15R (Capintec Inc., Ramsey, NJ, USA) have been published elsewhere^32^. The ^124^I solution for the spheres was taken from a stock solution, whereas the radioactivity for the cavity (background activity) was directly added to the water-filled phantom. Both the cavity and the stock solutions contained non-radioactive iodine to prevent adsorption on the phantom walls.

The initial activity concentration (AC) in the spheres and the background was derived from published thyroid cancer patient data^31^. (To avoid confusion, the ACs given in the text are all prepared ACs, that is, the AC derived from dose calibrator measurements.) Using the ACs from a total of 89 lymph node metastases and scaling the ACs to a commonly used administered ^124^I activity of 37 MBq, the statistics—expressed in mean (median) ± standard deviation (minimum–maximum)—of the actual ACs (after partial-volume-effect correction of the imaged ACs) was 155 kBq/mL (72 kBq/mL) ± 186 kBq/mL (1.6–691 kBq/mL). The AC ratio of lesion to background (signal-to-background)  was 1075 (346) ± 2069 (17–15,538), respectively. Corresponding numerical values for lung metastases and bone metastases are presented in the Supplemental Material (Supplemental Table S2). To simulate tumours under increasing challenging conditions, the initial (prepared) sphere AC was 25 kBq/mL and a signal-to-background ratio of 20:1 was used (values close to the minimum ranges of the evaluated DTC metastases). Lower sphere ACs were realised by 9 sequential PET acquisitions (temporal distance between each acquisition about one ^124^I half-life) and ranged from 25 kBq/ml down to 0.25 kBq/mL. The individual sphere and background ACs for both scanners at different starting times of PET image acquisition are shown in Table 1.

**Table 1:** Calculated sphere and background activity concentrations for both PET systems.

Number of measurement	Biograph Vision PET/CT system			Biograph mCT PET/CT system		
	Temporal distance to measurement 1 (h)	Sphere activity concentration (kBq/ml)	Background activity concentration (kBq/ml)	Temporal distance to measurement 1 (h)	Sphere activity concentration (kBq/ml)	Background activity concentration (kBq/ml)
1	0	24.99	1.24	0	24.85	1.23
2	67.0	15.72	0.78	67.0	15.63	0.78
3	161.6	8.17	0.41	161.7	8.13	0.40
4	233.8	4.83	0.24	237.7	4.93	0.24
5	331.4	2.53	0.13	331.0	2.51	0.12
6	402.5	1.37	0.07	420.1	1.54	0.08
7	498.4	0.80	0.04	497.1	0.79	0.04
8	571.1	0.43	0.02	588.4	0.48	0.02
9	666.6	0.26	0.01	659.9	0.25	0.01

### Phantom PET acquisition and its image reconstruction

#### PET acquisition

The phantom measurements were all one-bed scans with the phantom placed centrally within the scanner’s field of view. The acquisition started with a CT scan (tube voltage of 120 kVp, tube current time product of 15 mAs). Thereafter, PET emission data were acquired for 30 min in list mode format. The total phantom measurements lasted 28 days, resulting in lower and more challenging sphere ACs.

#### Image reconstruction

Both scanners allow for iterative image reconstruction algorithms. Image reconstructions were performed using (three-dimensional) ordinary Poisson ordered-subsets expectation maximization (OSEM), with TOF reconstruction alone (OSEM-TOF), or with both TOF and point spread function (PSF) modelling (OSEM-TOF+PSF). All PET data were reconstructed using our clinically standard reconstruction protocols that were optimized for quantitative ^124^I imaging^21,33^ and are listed in Table 2. All data were corrected for scatter, randoms, attenuation, dead time, decay, and normalization. In addition, for all PET systems, a prompt gamma coincidence correction method is by default implemented in the PET reconstruction algorithm for radionuclides emitting prompt gammas such as ^124^I.

**Table 2:** Overview of the image reconstruction parameters for both PET systems.

Iterative reconstruction	Scanner type	Iterations × subsets	Gauss filter (mm)	Matrix size	Voxel size (mm^3^)
OSEM	Vision	10 × 5	2	440 × 440	1.65 × 1.65 × 2.00
	mCT	3 × 24	2	400 × 400	2.04 × 2.04 × 2.00
OSEM-TOF	Vision	4 × 5	2	440 × 440	1.65 × 1.65 × 2.00
	mCT	2 × 21	2	400 × 400	2.04 × 2.04 × 2.00
OSEM-TOF+PSF	Vision	4 × 5	2	440 × 440	1.65 × 1.65 × 2.00
	mCT	2 × 21	2	400 × 400	2.04 × 2.04 × 2.00

#### Phantom data analysis

To quantify the detectability improvements by the SiPM-based system, the size-dependant MDA was used as metric^34^. To estimate the MDA, three steps were performed. First, all PET images were evaluated in a human observer study to assess the visual detectability of the small spheres. Second, the signal-to-noise ratio (SNR) of all spheres was estimated. Third, visual detectability and SNR were correlated to define the threshold SNR that indicates visual detectability (separately for OSEM, OSEM-TOF, and OSEM-TOF+PSF reconstructed images). The MDA was defined as the AC at the threshold SNR. In the following, the three steps are outlined in detail.

#### Human observer study

All data were interpreted by five nuclear medicine physicians (DK, MS, PS, CR, MW). The (lesion) detectability of each sphere was scored on an established 3-point scale differentiating between 0 = “not observed”, 1 = “observed but comparable to noise” or 2 = “observed”^34,35^. As an adaption of the previously published method by Øen et al.^35^, a sphere was rated as detected, if its sum score was ≥ 5.

#### Signal-to-noise ratio

The SNR of each sphere was determined for all evaluated data set (PET scanner, emission time and image reconstruction algorithm) using the following definition:

$$SNR= \frac{\left|\overline{{AC }_{sphere}} - \overline{{AC }_{background}}\right|}{{\sigma }_{background}},$$

with $\overline{{AC }_{sphere}}$ defined as mean sphere AC, $\overline{{AC }_{background}}$ as mean background AC, and ${\sigma }_{background}$ as standard deviation of ${AC}_{background}$.

Sphere ACs were evaluated in spherical volumes-of-interests (VOIs) of diameters that matched the real diameters of the small spheres (Fig. 1A). For positioning, the co-registered CT data were used; VOI positions were locally optimised in the 30-min emission time OSEM-TOF+PSF images with respect to a maximal mean sphere AC. The background AC was measured in 35 circular regions of interests of 20-mm diameter (representing twice the diameter of the largest sphere), which were positioned in the same transversal plane as the centres of the spherical VOIs (Fig. 1B).

#### Minimum detectable activity

The SNR distributions of detected and undetected spheres were visually analysed in a histogram plot to determine a threshold SNR indicating visibility^34,35^; the evaluation was separately performed for the different reconstruction algorithms. For each sphere, the SNR was analysed as a function of the AC. To reduce the influence of image noise, a cubic spline regression analysis was performed as previously described^36^. The MDA value was calculated as the AC at the threshold SNR.

### Activity concentration thresholds reflecting response levels to radioiodine therapy

Under certain assumptions, the actual ^124^I AC in the lesions can be used to predict the level of response to radioiodine therapy^11^. The key quantity is the absorbed (radiation) dose to the lesion. In the radioiodine treatment of lymph node metastases, a target dose of > 85 Gy reaches a high response of 98%, a target dose between 35 and 85 Gy a medium response of about 20%, whereas for a value below 35 Gy a therapeutic effect is unlikely^37^. Thus, the lesions can be categorized in a low, a medium, and a high absorbed dose group, according to the likelihood of success of radioiodine therapy. For small lesion sizes, the absorbed dose (*D*) mainly arises from beta-particle irradiation form ^131^I. Using the “medical internal radiation dose” (MIRD) formalism and spherical tumour model, the (average) self-absorbed (*D*) after administration of a therapeutic activity ($A_{0}^{{\text{I - 131}}}$) can be written in the following numerical value equation^38^:

$$\left( {{D \mathord{\left/ {\vphantom {D {{\text{Gy}}}}} \right. \kern-\nulldelimiterspace} {{\text{Gy}}}}} \right) = 3.809 \cdot \left( {{{C_{0}^{{\text{I-124}}} } \mathord{\left/ {\vphantom {{C_{0}^{{\text{I-124}}} } {{\text{kBq mL}}^{-1} }}} \right. \kern-\nulldelimiterspace} {{\text{kBq mL}}^{-1} }}} \right) \cdot \left( {{{T_{{\text{I-131}}}^{{{\text{eff}}}} } \mathord{\left/ {\vphantom {{T_{{\text{I-131}}}^{{{\text{eff}}}} } {\text{d}}}} \right. \kern-\nulldelimiterspace} {\text{d}}}} \right) \cdot \frac{{\left( {{{A_{0}^{{\text{I-131}}} } \mathord{\left/ {\vphantom {{A_{0}^{{\text{I-131}}} } {{\text{GBq}}}}} \right. \kern-\nulldelimiterspace} {{\text{GBq}}}}} \right)}}{{\left( {{{A_{0}^{{\text{I-124}}} } \mathord{\left/ {\vphantom {{A_{0}^{{\text{I-124}}} } {{\text{MBq}}}}} \right. \kern-\nulldelimiterspace} {{\text{MBq}}}}} \right)}}$$

Its derivation depends on several assumptions: (a) In the case of small lesions ranging from 3.7 to 10 mm, the absorbed dose essentially arises from self-irradiation by the beta particles of ^131^I. (b) In addition, particle-range effects due to the limited mean beta-particle range of ^131^I, even for the smallest sphere of 3.7 mm, can be neglected, as the percentage fraction of energy deposited within this lesion is still 90%^39^. (c) After administration of diagnostic activity ($A_{0}^{{\text{I-124}}}$), an instant ^124^I AC ($C_{0}^{{\text{I-124}}}$) and a monoexponential clearance with an effective half-life ($T_{{\text{I-131}}}^{{{\text{eff}}}}$) are assumed. Of note, the instant ^124^I AC equals the prepared sphere AC in our phantom setup. The equation given above was used to calculate the AC thresholds for the different absorbed lesion groups.

### Patient investigations

PET images of two thyroid cancer patients are presented to visualise the effects of the application of SiPM-based ^124^I PET. The detailed patient characteristics are: Patient #1: papillary thyroid cancer, male, 68 years, TNM: T3N1M1, unstimulated Tg: not measurable, Tg antibodies: 273 IU/mL, 1 previous radioiodine therapy, cumulative activity of ^131^I: 3.7 GBq. Patient #2: poorly differentiated thyroid cancer, male, 58 years, TNM: T3N0M1, unstimulated Tg: 1765 ng/ml, Tg antibodies: not measurable, 4 previous radioiodine therapies, cumulative activity of ^131^I: 21.5 GBq. Patient #1 underwent PET/CT acquisition on the mCT 18.1 h after oral application of 37.9 MBq of ^124^I, the PET/CT scan of patient #2 was started 17.4 h after application of 37.5 MBq of ^124^I on the mCT. Approximately 1 h after the acquisition on the mCT, PET/CT data were acquired on the Vision. Whole-body PET/CT data were acquired from head to thigh using 5–8 bed positions. Serum thyroid stimulating hormone level stimulation (≥ 30 mU/L) was achieved by levothyroxine withdrawal. The same PET protocol as described above for the phantom PET acquisition was used; the emission time was 4 min per bed position for each scanner type. PET/CT scans started with a whole-body spiral CT in low-dose technique without application of contrast agent (tube voltage of 120 kVp, tube current time product of 15 mAs, beam pitch of 1.0, and slice width of 5 mm). The patients gave written informed consent. The presentation of the patient examples was performed in accordance with the Declaration of Helsinki and approved by the institutional ethics committee (University of Duisburg-Essen, medical faculty, ethics protocol number 20-9203-BO).

### Software

PET data analysis was performed using PMOD 4.2 (PMOD Technologies LLC, Zurich, Switzerland). OriginPro 2019b (OriginLab, Northampton, Massachusetts, USA) and MATLAB R2019b (MathWorks, Natick, Massachusetts, USA) were used for data handling. Cubic spline regression analyses were performed using MATLAB R2019b.

## Results

In the phantom study, a total of 216 images (for each scanner type 9 ACs, 4 emission time durations for a single bed position, and 3 reconstruction algorithms) were analysed. As each image contains six spheres, a total of 1296 data points were evaluable. For reasons of clarity, only selected data are presented in the following section. The complete evaluated data are shown as Supplemental Material. In the following, all indicated emission times refer to a single bed position.

### Human observer study

Table 3 summarises the results of the human observer study; for each reconstruction algorithm, AC and reconstruction algorithm, the diameter of the smallest detected sphere is indicated. The data show a shift towards a smaller size of the smallest detected sphere from the mCT to the Vision, from OSEM to OSEM-TOF and from OSEM-TOF to OSEM-TOF+PSF image reconstruction, and from shorter to longer emission times (Table 3).

**Table 3:** Human observer study results—smallest detected sphere size (in mm) for each scanner type, reconstruction algorithm and emission time.

Sphere activity concentration (kBq/ml)	Scanner	OSEM				OSEM-TOF				OSEM-TOF+PSF			
		30 min	16 min	8 min	4 min	30 min	16 min	8 min	4 min	30 min	16 min	8 min	4 min
24.99	Vision	4.8	4.8	6.5	6.5	3.7	3.7	4.8	4.8	3.7	3.7	4.8	4.8
24.85	mCT	4.8	4.8	6.5	7.7	4.8	4.8	6.5	6.5	4.8	4.8	4.8	6.5
15.72	Vision	4.8	6.5	6.5	6.5	3.7	4.8	4.8	4.8	3.7	4.8	4.8	4.8
15.63	mCT	6.5	6.5	6.5	7.7	4.8	6.5	6.5	7.7	4.8	4.8	6.5	6.5
8.17	Vision	6.5	6.5	6.5	7.7	4.8	6.5	6.5	6.5	4.8	4.8	4.8	4.8
8.13	mCT	6.5	7.7	9.7	9.7	6.5	6.5	7.7	7.7	4.8	4.8	6.5	7.7
4.83	Vision	4.8	6.5	7.7	8.9	3.7	4.8	6.5	7.7	3.7	4.8	6.5	6.5
4.93	mCT	6.5	7.7	–	–	6.5	6.5	8.9	–	6.5	6.5	6.5	8.9
2.53	Vision	6.5	7.7	8.9	–	6.5	6.5	6.5	7.7	4.8	6.5	6.5	6.5
2.51	mCT	7.7	–	–	–	7.7	8.9	–	–	6.5	6.5	7.7	–
1.37	Vision	8.9	9.7	–	–	6.5	6.5	7.7	–	4.8	6.5	7.7	7.7
1.54	mCT	–	–	–	–	8.9	–	–	–	6.5	6.5	–	–
0.80	Vision	8.9	9.7	–	–	6.5	6.5	7.7	–	6.5	6.5	6.5	–
0.79	mCT	–	–	–	–	7.7	–	–	–	6.5	7.7	–	–
0.43	Vision	–	–	–	–	7.7	8.9	–	–	6.5	7.7	–	–
0.48	mCT	–	–	–	–	–	–	–	–	8.9	–	–	–
0.26	Vision	–	–	–	–	8.9	8.9	–	–	8.9	8.9	–	–
0.25	mCT	–	–	–	–	–	–	–	–	–	–	–	–

**Figure 2:**
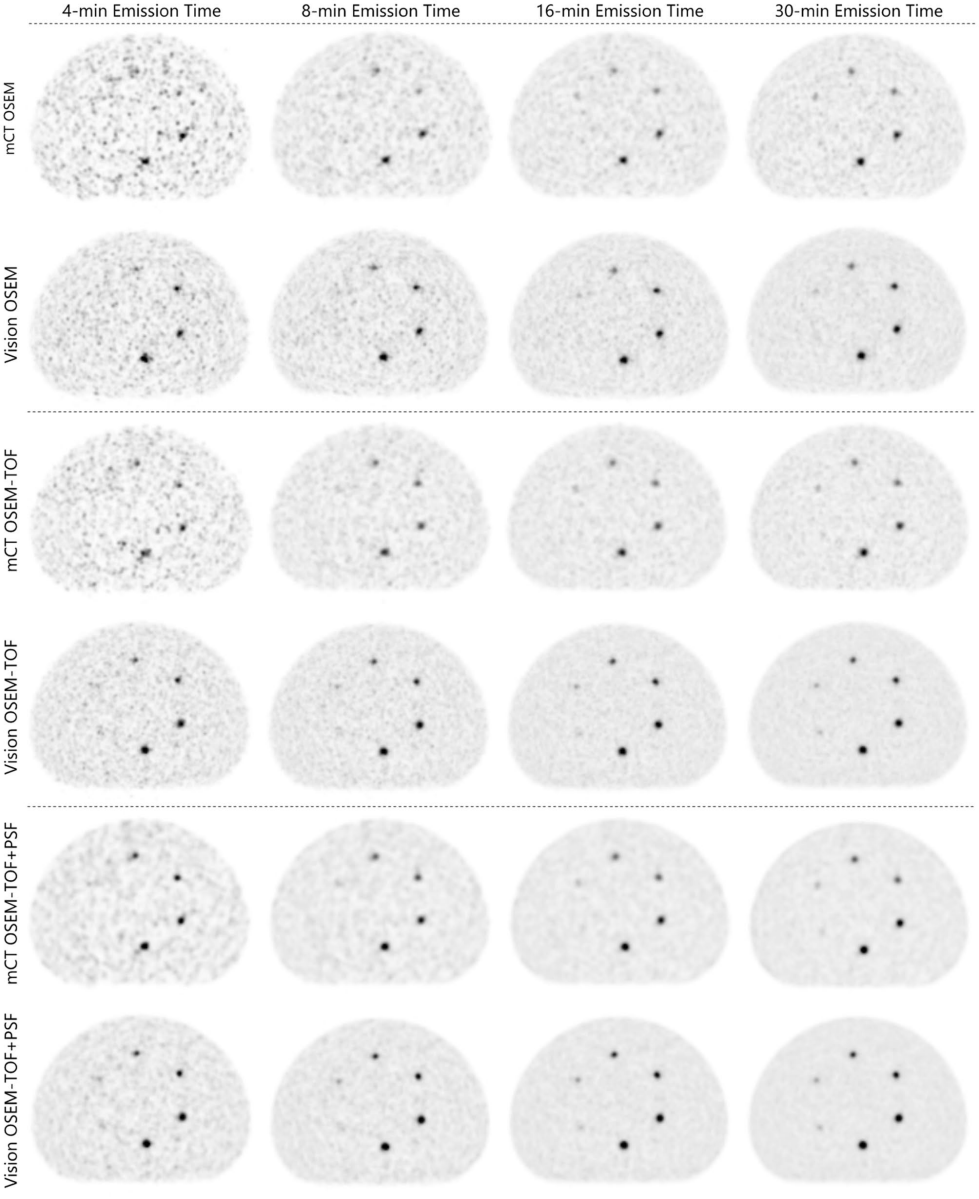
Evaluated PET images at the first imaging time point (sphere activity concentration ~ 25 kBq/ml). The position of the small spheres inside the phantom is presented in Fig. 1). Sphere diameters are depicted in Fig. 1A.

For example, the smallest sphere (3.7-mm diameter) was, at the highest AC, solely detectable on the Vision, using long emission times of ≥ 16 min, and using OSEM-TOF or OSEM-TOF+PSF image reconstruction. The second smallest sphere (4.8-mm diameter) was, at the highest AC and using OSEM-TOF or OSEM-TOF+PSF image reconstruction, detectable on the Vision at an emission time of ≥ 4 min, whereas on the mCT an emission time of ≥ 8 min was necessary (Fig. 2 and Table 3).

### Signal-to-noise ratio

**Figure 3:**
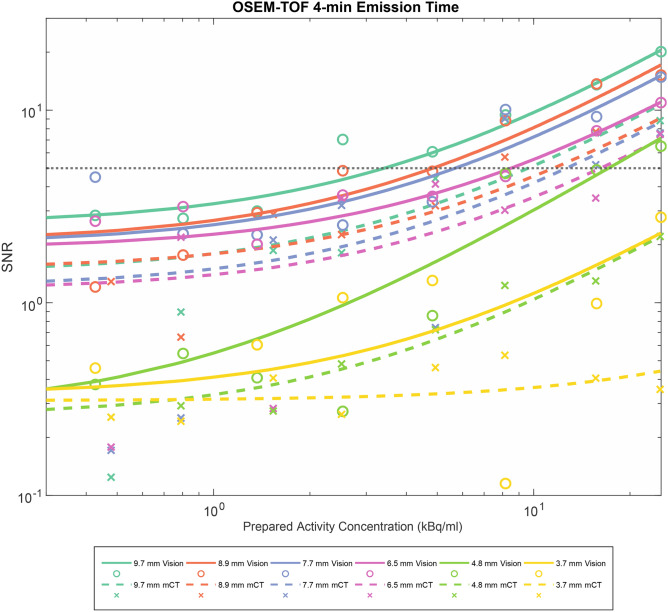
SNR as a function of the AC for all evaluated spheres for a clinical standard emission protocol (OSEM-TOF, 4-min emission time). A horizontal line at SNR = 5 indicates the threshold for visual detectability in OSEM-TOF reconstructed images.

The SNR shifted to higher values from the mCT to the Vision, from OSEM to OSEM-TOF and from OSEM-TOF to OSEM-TOF+PSF reconstructed images, from shorter to longer emission times, and from smaller to larger sphere sizes. For an acquisition comparable to our clinical standard PET protocol (4-min emission time, OSEM-TOF image reconstruction), the SNR is presented as a function of the AC for all sphere sizes in Fig. 3. Similar curves for all other imaging modalities are shown in the Supplemental Material.

**Figure 4:**
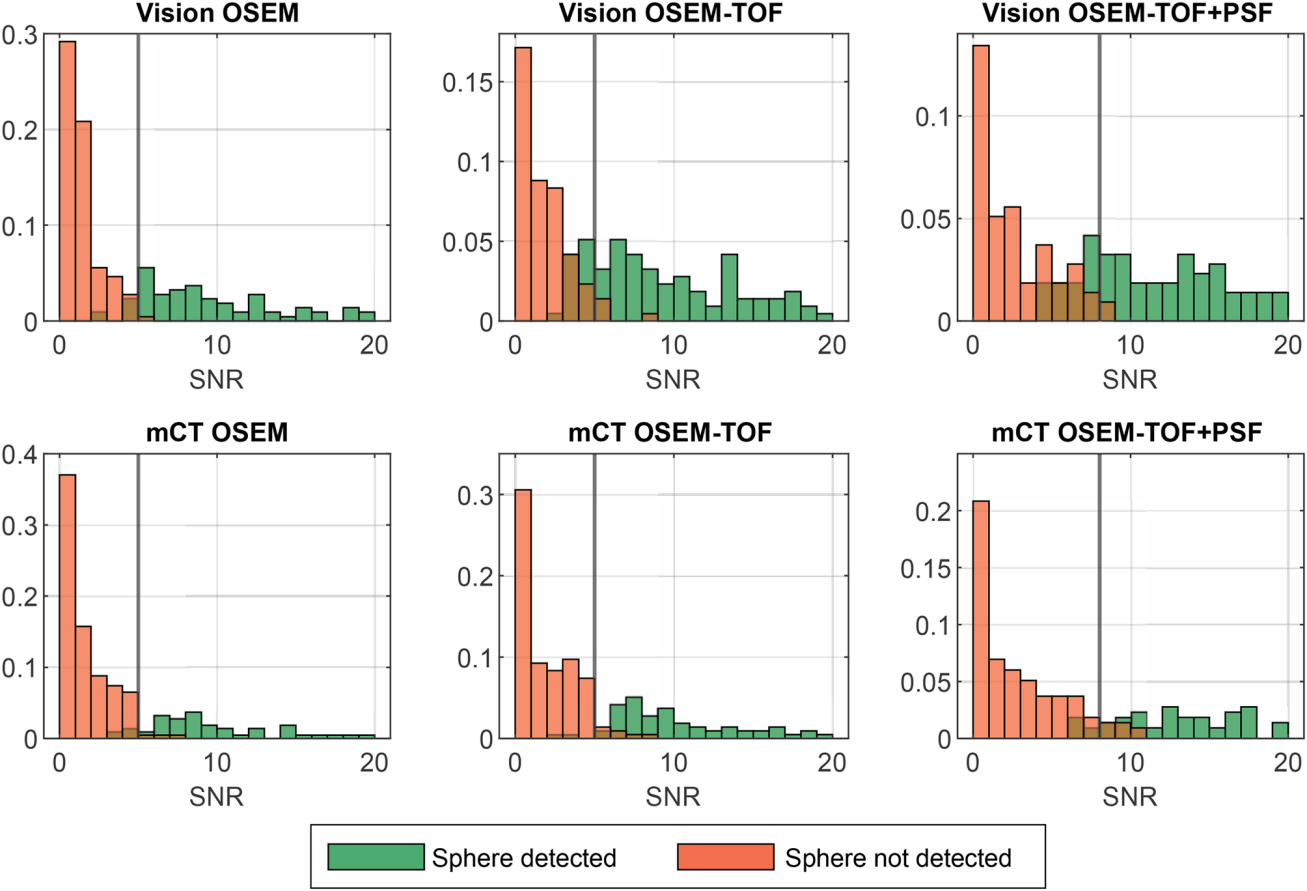
Normalised histograms of the SNR of detected and undetected spheres in the human observer study. Vertical lines at SNR = 5 for OSEM and OSEM-TOF image reconstruction, and SNR = 8 for OSEM-TOF+PSF image reconstruction, respectively, indicate the threshold that was derived to define a lesion as detectable in the mathematical model. For clarity purposes, SNR values > 20 are not displayed. The mixed organge-brown color arises from superposition of orange and green.

Histograms of the SNR of all detected and undetected spheres are shown in Fig. 4. For OSEM and OSEM-TOF reconstructions, a threshold value to indicate detectability of SNR ≥ 5, for OSEM-TOF+PSF, a value of SNR ≥ 8 was derived. These SNR threshold values were consistent with the commonly applied Rose criterion (originally derived from a theoretical contemplation of quantum effects in the visual process) that assumes a threshold SNR of 5 to define a detectable object^40^. The slightly higher threshold for the OSEM-TOF+PSF reconstructed images may be explained by a lower image noise that is a known phenomenon for PSF-reconstructed PET images^41^. In our data, the mean standard deviation of the background AC was lower in the OSEM-TOF+PSF reconstructed images. For example, for the 30-min emission time at the highest AC the ratios of the mean standard deviation of the background AC for OSEM-TOF+PSF to OSEM-TOF were 0.49 (mCT) and 0.61 (Vision). The respective ratios for OSEM-TOF+PSF to OSEM were 0.40 (mCT) and 0.44 (Vision).

### Minimum detectable activity

**Figure 5:**
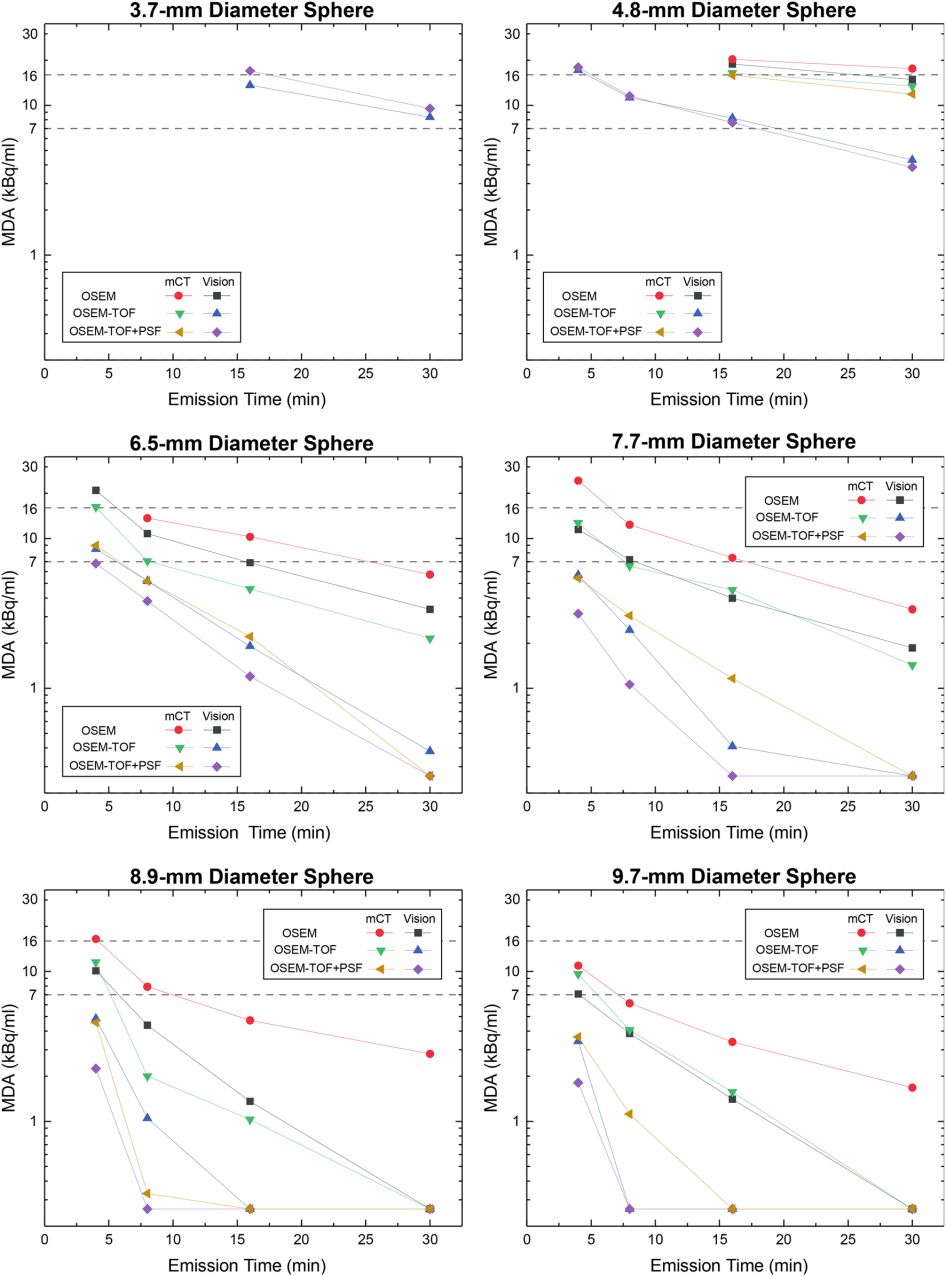
Semi-logarithmic representation of the MDA for both PET/CT systems as a function of the emission time for all spheres and image reconstructions. Horizontal lines at a MDA of 7 kBq/mL and at MDA of 16 kBq/mL are the dividing lines for the low, medium, and high absorbed dose lesion groups. Data points of a MDA < 0.26 kBq/ml were set to 0.26 kBq/ml meaning that the respective sphere was detected at every AC in this study.

For each sphere, reconstruction algorithm and PET scanner, the minimum detectable activity was separately calculated using the SNR threshold values derived from Fig. 4 (see above). Considering, for instance, the largest sphere (9.7 mm), the MDA is 1.8 kBq/ml on the Vision and 3.7 kBq/ml on the mCT at 4-min emission time and using OSEM-TOF+PSF reconstructed images (see Fig. 5, numbers are given in Supplemental Table S3 in the Supplemental Material). For the smallest sphere (3.7 mm), the MDA is 17.0 kBq/ml on the Vision at 16-min emission time and using OSEM-TOF+PSF reconstructed images; this sphere was not detected on the mCT.

In the relative comparison, the MDA decreased from the mCT to the Vision, from OSEM to OSEM-TOF and from OSEM-TOF to OSEM-TOF+PSF image reconstruction, with increasing emission time, and with increasing sphere size (Fig. 5). Across all evaluated emission times and reconstruction algorithms, the (overall) mean MDA ratio of Vision to mCT is 0.52 ± 0.18. Comparing the different image reconstruction algorithms, the mean MDA ratio is 0.61 ± 0.17 (OSEM), 0.41 ± 0.17 (OSEM-TOF), and 0.53 ± 0.15 (OSEM-TOF + PSF), respectively. No dependency of the mean MDA ratio on the emission time (0.47 ± 0.25 for 30-min, 0.59 ± 0.23 for 16-min, 0.59 ± 0.16 for 8-min, and 0.53 ± 0.12 for 4-min emission time, respectively) or on the sphere size is observed (Table 4). The MDA for all evaluated data points and the MDA ratio separately for each combination of emission time and image reconstruction algorithm is shown as Supplemental Material.

**Table 4:** MDA-Ratios between the Vision and the mCT for each sphere size, reconstruction algorithm, and emission time.

Sphere diameter (mm)	OSEM				OSEM-TOF				OSEM-TOF+PSF				All reconstructions
	30-min	16-min	8-min	4-min	30-min	16-min	8-min	4-min	30-min	16-min	8-min	4-min	All emission times (mean ± SD)
3.7	–	–	–	–	–	–	–	–	–	–	–	–	–
4.8	0.84	0.93	–	–	0.32	0.50	–	–	0.33	0.48	–	–	0.56 ± 0.26
6.5	0.59	0.67	0.79	–	0.18	0.42	0.74	0.53	–	0.54	0.74	0.76	0.60 ± 0.19
7.7	0.56	0.54	0.59	0.47	–	0.09	0.38	0.45	–	–	0.35	0.58	0.45 ± 0.16
8.9	–	0.29	0.55	0.61	–	–	0.53	0.42	–	–	–	0.49	0.48 ± 0.12
9.7	–	0.42	0.63	0.65	–	–	–	0.36	–	–	–	0.49	0.51 ± 0.13
All spheres (mean ± SD)	0.66 ± 0.16	0.57 ± 0.25	0.65 ± 0.11	0.58 ± 0.10	0.25 ± 0.11	0.34 ± 0.22	0.55 ± 0.19	0.44 ± 0.08	0.33 ± 0	0.51 ± 0.05	0.54 ± 0.28	0.58 ± 0.13	0.52 ± 0.18

No values are indicated, if the MDA was outside the range that can be evaluated within this investigation (< 0.26 kBq/ml or > 25 kBq/ml) for one or both examined PET/CT systems.

### Correlation between minimum detectable activity and emission time

For validation, we determined the MDA ratio of shorter to longer emission time for all spheres for which exact MDA values for two emission times that differed by a factor of two were available (n = 41). The mean ± standard deviation ratio of shorter to longer emission time was 0.48 ± 0.15. This confirms that, in a first approximation, the MDA correlates linearly with the emission time.

### Activity concentration thresholds for the absorbed lesion groups

In the calculation, a mean effective ^131^I half-life of approximately 3.5 days was used, a value that was derived from 52 lymph node metastases^42^. Moreover, an applied ^124^I activity of 37 MBq (based on our clinically established dosimetry protocol) and a maximum single ^131^I therapeutic activity of 15 GBq applied at our department^11,43^ were selected. A threshold AC of 7 kBq/ml was calculated to achieve a lesion dose of 35 Gy and a threshold AC of 16 kBq/ml is required to achieve a lesion dose of 85 Gy. Thus, the three lesion groups exhibit AC ranges of AC < 7 kBq/ml for the low absorbed dose group, 7 ≤ AC ≤ 16 kBq/ml for the medium absorbed dose group, and AC > 16 kBq/ml for the high absorbed dose group. These three groups are separated by horizontal lines in Fig. 5.

Of note, transferring above model calculations to clinical lesion dosimetry, several contributions limiting the accuracy of absorbed doses are to be considered. The largest uncertainties in small lesion absorbed dose estimates, as investigated in this phantom study, remain the volume determinations. Assuming an uncertainty in diameter of ± 1 mm in each dimension, the maximum error uncertainties, for the smallest (3.7 mm) and for the largest (9.7 mm) investigated spheres are approximately 80% and 30%, respectively. Other error contributions are, for instance, related to parameterisation of the time-activity curves and activity concentration measurements.

### Patient examples

**Figure 6:**
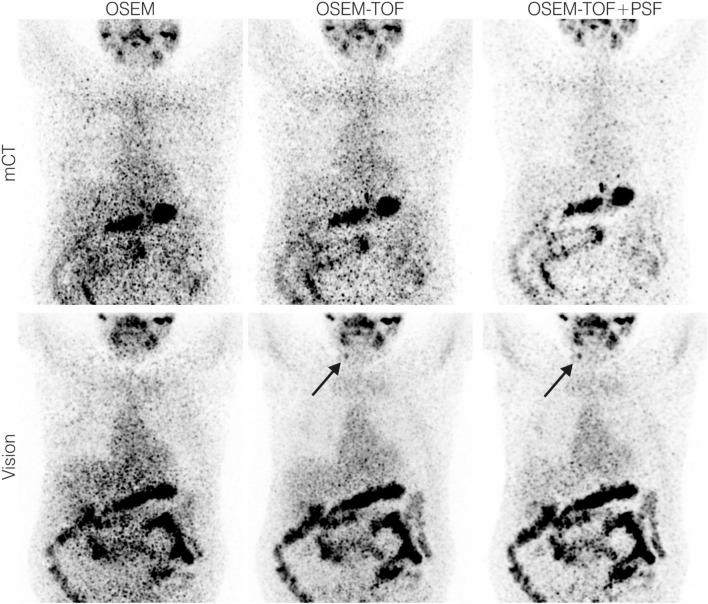
Maximum intensity projection PET images of exemplary patient data set 1 acquired using our clinical PET protocol (4-min emission time per bed position, image acquisition approximately 1 day after application of 37.9 MBq of ^124^I). The black arrow indicates an additional cervical lymph node metastasis detected on the SiPM-based system.

**Figure 7:**
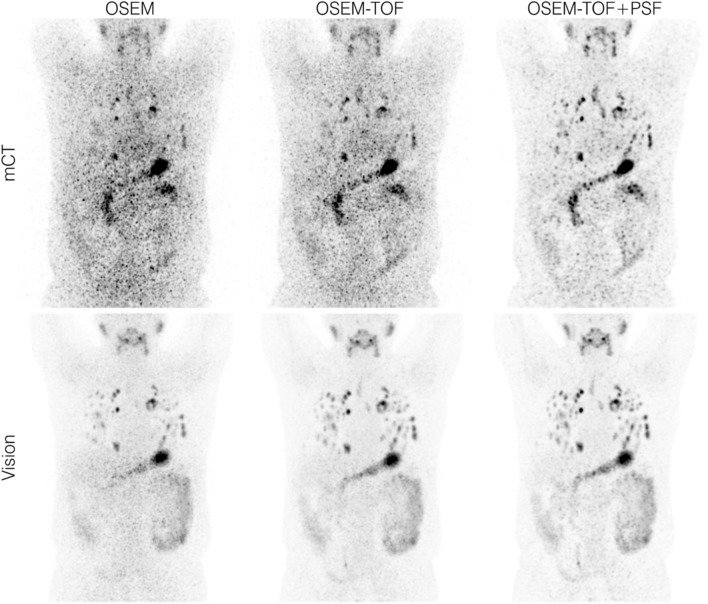
Maximum intensity projection PET images of exemplary patient data set 2 acquired using our clinical PET protocol (4-min emission time per bed position, image acquisition approximately 1 day after application of 37.5 MBq of ^124^I). The size range of the detected lung lesions was 4–24 mm in diameter.

In patient 1, an additional ^124^I-avid cervical lymph node metastasis was detected (Fig. 6). In this patient three other lymph node metastases were detected also on the mCT and therapy management (watchful waiting) was not altered. As shown in Fig. 7, multiple additional ^124^I-avid pulmonary metastases are observed in patient 2. In this patient, therapy management was chosen to watchful waiting due to the presence of other radioiodine-negative metastases. Visually, image quality and detectability increase, and image noise decreases from the mCT to the Vision, and from OSEM to OSEM-TOF and OSEM-TOF to OSEM-TOF+PSF reconstructed images.

## Discussion

In the comparison between the SiPM- and PMT-based systems, a qualitative and a quantitative detectability analysis were performed. In the qualitative human observer study, the detectability was higher on the Vision (Table 3, Figs. 3 and 4). The clinical PET image exemplified the benefits of the SiPM-based PET system (Fig. 6 & Fig. 7). In the quantitative comparison of the MDA (which is a scanner-specific metric under fixed acquisition conditions), the Vision outperformed the mCT in all examined imaging conditions (Table 4); the overall mean MDA ratio (Vision to mCT) was 0.52 ± 0.18. Both an improved detectability^21,44^ and an increased SNR^45^ for small spheres were previously observed for SiPM-based PET/CT systems in studies using ^18^F-FDG.

In agreement with a previous study by Beijst et al.^36^ who evaluated slightly larger spheres (≥ 10 mm) and only used the mCT, the MDA decreased from OSEM to OSEM-TOF and from OSEM-TOF to OSEM-TOF+PSF image reconstruction and with increasing sphere size (Fig. 5). Of note, the MDA ratio did not correlate with the emission time or the sphere size (Table 4). The MDA ratio was slightly improved for OSEM-TOF and OSEM-TOF+PSF image reconstructions (Table 4), a possible explanation may be the improved timing resolution of the Vision (214 ps for the Vision versus 540 ps for the mCT). The improved timing resolution is particularly beneficial for the typically noisy ^124^I PET images^22^ that are limited by a low positron branching ratio and low administered activities.

Based on these phantom data, we attempt to predict the response to radioiodine therapy of those lesions that are only identified on the Vision (Fig. 5). The following considerations refer to the image reconstruction with the best detectability, i.e., OSEM-TOF or OSEM-TOF+PSF on the Vision and OSEM-TOF+PSF on the mCT, respectively. Moreover, lesions are assigned to three groups by their AC (low, medium, and high absorbed dose groups) that correlate with a possible success of radioiodine therapy. The respective AC thresholds were based on patient data, who underwent imaging after application of 37 MBq of ^124^I (according to our standard clinical PET protocol), an observed mean effective ^131^I half-life of 3.5 days for lymph node metastases, and maximum single ^131^I therapeutic activity of 15 GBq. Also, the selected sphere sizes reflect the typical size range for cervical lymph nodes^30,31^.

A striking feature is that the AC thresholds indicating treatment response to radioiodine therapy fall within the MDA range of the PET/CT devices under clinical acquisition conditions. Thus, the detectability of a treatable lesion can depend on the emission time and a separate evaluation of different-sized lesions is mandatory. One major consequence of the detailed analysis is that for spheres ≥ 6.5 mm in diameter, lesions from each group could be identified on both PET/CT systems using a standard clinical emission time of 4-min per bed position. Regarding the second smallest sphere (4.8-mm diameter) at 4-min emission time per bed position, only lesions belonging to the high absorbed dose group would be detectable on the Vision. To identify lesions belonging to all absorbed dose groups, an increased emission time of 16 min per bed position would be necessary. On the mCT, a 16-min emission time per bed position would be necessary to detect lesions from the high absorbed dose group, lesions from the low and medium groups would not be detectable even at an emission time of 30 min per bed position. For the smallest sphere (3.7-mm diameter), lesions from the high absorbed dose group would be detectable on the Vision at 16-min and from all groups at 30-min emission time per bed position. Of note, the smallest sphere was not detectable on the mCT under any examined condition.

The examined conditions also cover the typical activity ranges of clinically detected lung and bone lesions (Supplemental Table S2 in the Supplemental Material). In the evaluated pulmonary metastasised DTC patient (patient 2), the size range of lung metastases (4–24 mm in diameter) indicates that the size range of small detectable pulmonary metastases is also represented in this study. We therefore suppose that the results can be projected to small lung metastases. Patients with even smaller disseminated pulmonary metastases (i.e., miliary pulmonary metastases that are detected in intratherapeutic ^131^I scintigraphy) can be challenging^46^, even for SiPM-based ^124^I PET/CT. A published method that may help to detect these lesions and can also be applied for SiPM-based systems is the quantitative analysis of the ^124^I activity concentration ratio of the lung to the background (L/B ratio)^46^. A detailed analysis of patient data may be warranted to evaluate the clinical benefit of the L/B ratio for SiPM based PET/CT systems.

Further consequences are related to “blind” radioiodine therapies and the results published by the THYROPET study^14^. The improved detectability using the Vision might allow for the additional identification of small lesions that could respond to radioiodine therapy. Hence, the number of patients subjected to “blind” radioiodine therapy might be reduced, thus reducing the risk of adverse effects in a scenario, in which a clinical benefit is unlikely. The data also suggest that a limited PET scanner sensitivity of previous-generation scanners can contribute to explain the discrepancies between ^124^I PET and intratherapeutic ^131^I imaging that were reported in previous studies including the prospective THYROPET study^13,14^. Specifically, the Biograph mCT that was used as example for a previous-generation PET/CT system in the present study was a high-end scanner at the time of the published results that reported false negative ^124^I PET findings^13,14^. In the previous studies, not only the mCT but also less sophisticated PET/CT systems, some without TOF modelling, were used^13,14^. Following our data, on the mCT without TOF modelling, for the 6.5-mm and 7.7-mm spheres, lesions belonging to the low and medium absorbed dose groups would not be identified.

The clinical benefit of additionally detected lesions may be limited by the presence of other metastases that are also visualised by older devices. In the two patients presented (Figs. 6 and 7), the additional lesions had no impact on therapy management. We recently performed a clinical evaluation of SiPM-based ^124^I PET/CT in patients with thyroglobulin levels in the low measurable range^21^. Additional lesions were detected in 2 of 10 patients; however, only in one patient the therapeutic concept was changed (begin of radioiodine therapy)^21^.

The results can also be used to discuss a clinical acquisition protocol that allows for the detection of therapeutically relevant lesions. Using a SiPM-based PET/CT system, OSEM-TOF or OSEM-TOF+PSF reconstructed images and an emission time of 8 min (per bed position), all spheres except the smallest 3.7-mm diameter sphere were detectable, a sphere size being at the lower end of typical lymph node metastases in the neck region^30^. For smaller lesions, an increased emission time could be necessary. However, the maximum emission time of a clinical scan is limited by patient condition and the availability of PET scanning time. A particularly increased emission time for single regions, e.g., the neck region that is mostly affected by DTC lymph node metastases^1^, appears to be feasible. Our results suggest a linear correlation between emission time and MDA. Alternatively, the applied amount of ^124^I could be increased to improve the detectability. According to published literature, up to 74 MBq of ^124^I are applied in clinical protocols^7^. The application of higher amounts of ^124^I may be critical due to thyroid stunning (i.e., diminished uptake of ^131^I after diagnostic scans), which has not been conclusively clarified yet for ^124^I^47^.

Besides the usage of SiPM-based PET/CT systems, the implantation of improved image reconstruction algorithms may increase the detectability of small lesions. Since SIPM-based systems will not probably be available in all centres within the next years, software improvements are particularly important because they can also be applied to existing PET/CT systems.

The study faces four limitations. First, the discussed clinical consequences are limited by the assumption of the administration of 37 MBq of ^124^I. However, this is a commonly applied activity in clinical protocols^21^. Second, in contrast to the phantom setting, real metastases can be inhomogeneous, of non-spherical geometry, their signal-to-background ratio can be variable, and their PET signal can be influenced by motions. Moreover, an abdominal phantom was used that could affect a projection of the results to the neck region. However, these limitations affect both examined PET systems. Third, the spheres are located at fixed positions; therefore, the human observer analysis is biased by prior knowledge. Fourth, the choice of reconstruction parameters was guided by the manufacturer’s recommendation; further MDA improvements could be achieved by an optimisation study (e.g., regarding number of iterations).

## Conclusion

The overall mean MDA for ^124^I was improved by a factor of 0.52 ± 0.18 for the Biograph Vision in comparison to the Biograph mCT. Under challenging conditions, all lesions with expected response to radioiodine therapy of ≥ 6.5 mm in diameter could be identified on both PET/CT systems using a standard clinical protocol. Lesions of smaller size—still with predicted response to radioiodine therapy—could be detected when applying a SiPM-based PET system at reasonable scan duration times. Further clinical studies are warranted to evaluate if negative ^124^I PET scans on a SiPM-based system can be sufficient to preclude patients from blind radioiodine therapy. In the future, the clinical application of total body PET systems can further reduce the MDA.

### Supplementary Information

Supplementary Information.
