# Supplementary material for: Silicon-photomultiplier-based PET/CT reduces the minimum detectable activity of iodine-124
Source: Sci Rep. 2021 Sep 1;11:17477. doi: 10.1038/s41598-021-95719-8 (PMC8410931; doi:10.1038/s41598-021-95719-8)
Supplement: Supplementary file 1 — Supplementary Information. [file 41598_2021_95719_MOESM1_ESM.pdf]

Supplementary Material to:

Silicon-Photomultiplier-Based PET/CT Reduces the Minimum Detectable Activity of Iodine-124

D. Kersting* [1,4], W. Jentzen[1,4], P. Fragoso Costa[1,4], M. Sraieb[1,4], P. Sandach[1,4], L. Umutlu[2,4], M. Conti[3], F. Zarrad[1,4], C. Rischpler[1,4], W. P. Fendler[1,4], K. Herrmann[1,4], and M. Weber[1,4]

[1] Department of Nuclear Medicine, University Hospital Essen, University of Duisburg-Essen, Essen, Germany

[2] Department of Diagnostic and Interventional Radiology and Neuroradiology, University Hospital Essen, University of Duisburg-Essen, Essen, Germany

[3] Siemens Medical Solutions USA, Inc., Knoxville, TN, United States

[4] German Cancer Consortium (DKTK, partner site Essen), Essen, Germany

Supplemental Figure S1

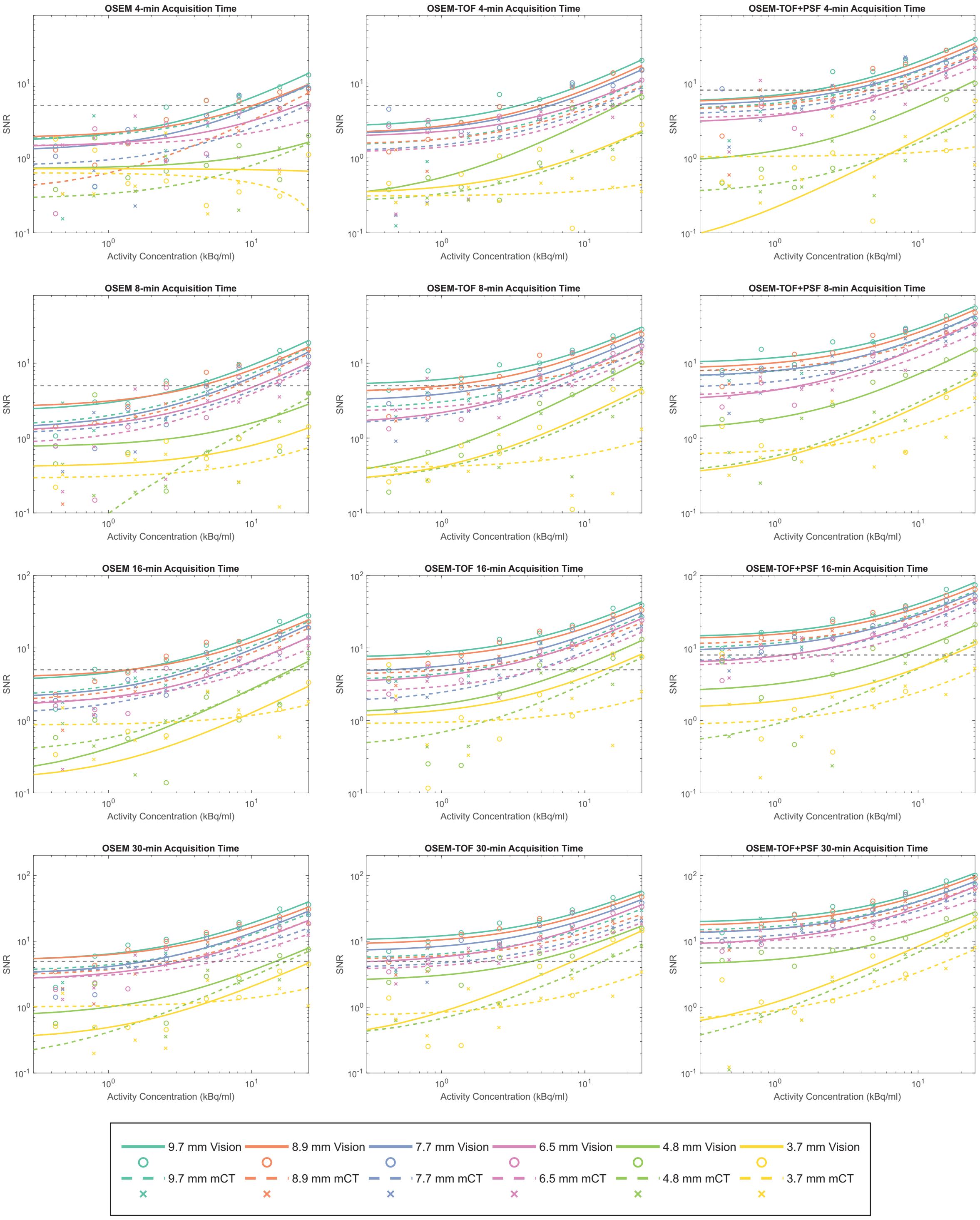

Figure 1: SNR as a function of the AC for all evaluated spheres, separately for all evaluated reconstruction algorithms and emission times. A horizontal line at SNR = 5 (OSEM and OSEM-TOF) or SNR = 8 (OSEM-TOF+PSF), respectively, indicates the threshold for visual detectability.

Supplemental Table S1: Scanner Specifications

Technical specifications for the Biograph mCT and the Biograph Vision. LSO = Lutetium Oxyorthosilicate.

	**Biograph mCT**	**Biograph Vision**
Detector material	LSO	LSO
Detector element dimension ($mm^{3)}$	4x4x20	3.2x3.2x20
Detector elements per block	13x13	16x16
Total number of detector elements	33,448	60,800
Signal readout	PMTs (2x2 per block)	SiPM (2x2 per block)
Axial FOV (cm)	21.8	26.3
Transaxial FOV (cm)	70	78
Plane spacing (mm)	2	1.65
Image planes	109	119
Coincidence time window (ns)	4.1	4.7
Energy window (keV)	435-650	435-585
Energy resolution (%)	11	9
System time resolution (ps)	540	210
NEMA sensitivity (kcps/MBq)	9.7	16.4

Supplemental Table S2: Activity Concentration Characterization for Different Metastases

Activity Concentrations (AC) and AC to background AC for different types of differentiated thyroid cancer metastases.

	Lymph node metastases	Lung metastases	Bone metastases
**Number of evaluated metastases**	89	34	61
**AC (kBq/ml)**			
mean	155	22	104
median	72	16	60
standard deviation	186	16	124
minimum-maximum	1.6–691	3.5-72	5.4-684
**Ratio AC to the background AC**			
mean	1075	91	659
median	346	38	232
standard deviation	2069	199	976
minimum-maximum	17–15538	11-1186	21-5167

Supplemental Table S3: MDA for each evaluated sphere size, PET/CT system, reconstruction algorithm and emission time.

Sphere Diameter (mm)	Scanner	OSEM				OSEM+TOF				OSEM+TOF+PSF			
		30-min	16-min	8-min	4-min	30-min	16-min	8-min	4-min	30-min	16-min	8-min	4-min
3.7	Vision	>25	>25	>25	>25	8.33	13.59	>25	>25	9.51	16.96	>25	>25
	mCT	>25	>25	>25	>25	>25	>25	>25	>25	>25	>25	>25	>25
4.8	Vision	14.86	18.87	>25	>25	4.32	8.22	11.26	17.16	3.87	7.67	11.52	17.96
	mCT	17.59	20.29	>25	>25	13.41	16.43	>25	>25	11.86	15.87	>25	>25
6.5	Vision	3.36	6.88	10.76	20.93	0.38	1.91	5.20	8.50	<0.26	1.20	3.82	6.80
	mCT	5.74	10.25	13.66	>25	2.15	4.60	7.05	16.19	<0.26	2.21	5.19	8.98
7.7	Vision	1.86	3.99	7.22	11.47	<0.26	0.41	2.45	5.70	<0.26	<0.26	1.06	3.15
	mCT	3.36	7.42	12.34	24.33	1.43	4.53	6.52	12.68	<0.26	1.16	3.05	5.43
8.9	Vision	<0.26	1.36	4.38	10.12	<0.26	<0.26	1.05	4.85	<0.26	<0.26	<0.26	2.25
	mCT	2.82	4.72	7.92	16.50	<0.26	1.03	2.00	11.53	<0.26	<0.26	0.33	4.57
9.7	Vision	<0.26	1.41	3.86	7.08	<0.26	<0.26	<0.26	3.42	<0.26	<0.26	<0.26	1.81
	mCT	1.68	3.39	6.14	10.95	<0.26	1.57	4.07	9.61	<0.26	<0.26	1.12	3.66
Ratio Vision/mCT Mean±SD		0.66 ± 0.16	0.57 ± 0.25	0.65 ± 0.11	0.58 ± 0.10	0.25 ± 0.11	0.34 ± 0.22	0.55 ± 0.19	0.44 ± 0.08	0.33 ± 0	0.51 ± 0.05	0.54 ± 0.28	0.58 ± 0.13
Number of evaluated spheres		3	5	4	3	2	3	3	4	1	2	2	4

Presented values in units of kBq/ml. MDA <0.26 kBq/ml: No MDA could be calculated, as the SNR was > cut-off value for visual detection for all ACs (meaning detection for every examined AC). MDA >25 kBq/ml: No MDA could be calculated, as the SNR was < cut-off value for all ACs (meaning no detection for any examined AC).

Supplemental Table S4: Human observer study results for the 3.7-mm sphere.

The human observer study results are separately presented for the examined PET/CT scanners, reconstruction algorithms and emission times. 1 = detected. 0 = undetected.

Sphere Activity Concentration (kBq/ml)	Scanner	OSEM				OSEM+TOF				OSEM+TOF+PSF			
		30 min	16 min	8 min	4 min	30 min	16 min	8 min	4 min	30 min	16 min	8 min	4 min
24.99	Vision	0	0	0	0	1	1	0	0	1	1	0	0
24.85	mCT	0	0	0	0	0	0	0	0	0	0	0	0
15.72	Vision	0	0	0	0	1	0	0	0	1	0	0	0
15.63	mCT	0	0	0	0	0	0	0	0	0	0	0	0
8.17	Vision	0	0	0	0	0	0	0	0	0	0	0	0
8.13	mCT	0	0	0	0	0	0	0	0	0	0	0	0
4.83	Vision	0	0	0	0	1	0	0	0	1	0	0	0
4.93	mCT	0	0	0	0	0	0	0	0	0	0	0	0
2.53	Vision	0	0	0	0	0	0	0	0	0	0	0	0
2.51	mCT	0	0	0	0	0	0	0	0	0	0	0	0
1.37	Vision	0	0	0	0	0	0	0	0	0	0	0	0
1.54	mCT	0	0	0	0	0	0	0	0	0	0	0	0
0.80	Vision	0	0	0	0	0	0	0	0	0	0	0	0
0.79	mCT	0	0	0	0	0	0	0	0	0	0	0	0
0.43	Vision	0	0	0	0	0	0	0	0	0	0	0	0
0.48	mCT	0	0	0	0	0	0	0	0	0	0	0	0
0.26	Vision	0	0	0	0	0	0	0	0	0	0	0	0
0.25	mCT	0	0	0	0	0	0	0	0	0	0	0	0

Supplemental Table S5: Human observer study results for the 4.8-mm sphere.

The human observer study results are separately presented for the examined PET/CT scanners, reconstruction algorithms and emission times. 1 = detected. 0 = undetected.

Sphere Activity Concentration (kBq/ml)	Scanner	OSEM				OSEM+TOF				OSEM+TOF+PSF			
		30 min	16 min	8 min	4 min	30 min	16 min	8 min	4 min	30 min	16 min	8 min	4 min
24.99	Vision	1	1	0	0	1	1	1	1	1	1	1	1
24.85	mCT	1	1	0	0	1	1	0	0	1	1	1	0
15.72	Vision	1	0	0	0	1	1	1	1	1	1	1	1
15.63	mCT	0	0	0	0	1	0	0	0	1	1	0	0
8.17	Vision	0	0	0	0	1	0	0	0	1	1	1	1
8.13	mCT	0	0	0	0	0	0	0	0	1	1	0	0
4.83	Vision	1	0	0	0	1	1	0	0	1	1	0	0
4.93	mCT	0	0	0	0	0	0	0	0	0	0	0	0
2.53	Vision	0	0	0	0	0	0	0	0	1	0	0	0
2.51	mCT	0	0	0	0	0	0	0	0	0	0	0	0
1.37	Vision	0	0	0	0	0	0	0	0	1	0	0	0
1.54	mCT	0	0	0	0	0	0	0	0	0	0	0	0
0.80	Vision	0	0	0	0	0	0	0	0	0	0	0	0
0.79	mCT	0	0	0	0	0	0	0	0	0	0	0	0
0.43	Vision	0	0	0	0	0	0	0	0	0	0	0	0
0.48	mCT	0	0	0	0	0	0	0	0	0	0	0	0
0.26	Vision	0	0	0	0	0	0	0	0	0	0	0	0
0.25	mCT	0	0	0	0	0	0	0	0	0	0	0	0

Supplemental Table S6: Human observer study results for the 6.5-mm sphere.

The human observer study results are separately presented for the examined PET/CT scanners, reconstruction algorithms and emission times. 1 = detected. 0 = undetected.

Sphere Activity Concentration (kBq/ml)	Scanner	OSEM				OSEM+TOF				OSEM+TOF+PSF			
		30 min	16 min	8 min	4 min	30 min	16 min	8 min	4 min	30 min	16 min	8 min	4 min
24.99	Vision	1	1	1	1	1	1	1	1	1	1	1	1
24.85	mCT	1	1	1	0	1	1	1	1	1	1	1	1
15.72	Vision	1	1	1	1	1	1	1	1	1	1	1	1
15.63	mCT	1	1	1	0	1	1	1	0	1	1	1	1
8.17	Vision	1	1	1	0	1	1	1	1	1	1	1	1
8.13	mCT	1	0	0	0	1	1	0	0	1	1	1	0
4.83	Vision	1	1	0	0	1	1	1	0	1	1	1	1
4.93	mCT	1	0	0	0	1	1	0	0	1	1	1	0
2.53	Vision	1	0	0	0	1	1	1	0	1	1	1	1
2.51	mCT	0	0	0	0	0	0	0	0	1	1	0	0
1.37	Vision	0	0	0	0	1	1	0	0	1	1	0	0
1.54	mCT	0	0	0	0	0	0	0	0	1	1	0	0
0.80	Vision	0	0	0	0	1	1	0	0	1	1	1	0
0.79	mCT		0	0	0	0	0	0	0	1	0	0	0
0.43	Vision	0	0	0	0	0	0	0	0	1	0	0	0
0.48	mCT	0	0	0	0	0	0	0	0	0	0	0	0
0.26	Vision	0	0	0	0	0	0	0	0	0	0	0	0
0.25	mCT	0	0	0	0	0	0	0	0	0	0	0	0

Supplemental Table S7: Human observer study results for the 7.7-mm sphere.

The human observer study results are separately presented for the examined PET/CT scanners, reconstruction algorithms and emission times. 1 = detected. 0 = undetected.

Sphere Activity Concentration (kBq/ml)	Scanner	OSEM				OSEM+TOF				OSEM+TOF+PSF			
		30 min	16 min	8 min	4 min	30 min	16 min	8 min	4 min	30 min	16 min	8 min	4 min
24.99	Vision	1	1	1	1	1	1	1	1	1	1	1	1
24.85	mCT	1	1	1	1	1	1	1	1	1	1	1	1
15.72	Vision	1	1	1	1	1	1	1	1	1	1	1	1
15.63	mCT	1	1	1	1	1	1	1	1	1	1	1	1
8.17	Vision	1	1	1	1	1	1	1	1	1	1	1	1
8.13	mCT	1	1	0	0	1	1	1	1	1	1	1	1
4.83	Vision	1	1	1	0	1	1	1	1	1	1	1	1
4.93	mCT	1	1	0	0	1	1	0	0	1	1	1	0
2.53	Vision	1	1	0	0	1	1	1	1	1	1	1	1
2.51	mCT	1	0	0	0	1	0	0	0	1	1	1	0
1.37	Vision	0	0	0	0	1	1	1	0	1	1	1	1
1.54	mCT	0	0	0	0	0	0	0	0	1	1	0	0
0.80	Vision	0	0	0	0	1	1	1	0	1	1	1	0
0.79	mCT	0	0	0	0	1	0	0	0	1	1	0	0
0.43	Vision	0	0	0	0	1	0	0	0	1	1	0	0
0.48	mCT	0	0	0	0	0	0	0	0	0	0	0	0
0.26	Vision	0	0	0	0	0	0	0	0	0	0	0	0
0.25	mCT	0	0	0	0	0	0	0	0	0	0	0	0

Supplemental Table S8: Human observer study results for the 8.9-mm sphere.

The human observer study results are separately presented for the examined PET/CT scanners, reconstruction algorithms and emission times. 1 = detected. 0 = undetected.

Sphere Activity Concentration (kBq/ml)	Scanner	OSEM				OSEM+TOF				OSEM+TOF+PSF			
		30 min	16 min	8 min	4 min	30 min	16 min	8 min	4 min	30 min	16 min	8 min	4 min
24.99	Vision	1	1	1	1	1	1	1	1	1	1	1	1
24.85	mCT	1	1	1	1	1	1	1	1	1	1	1	1
15.72	Vision	1	1	1	1	1	1	1	1	1	1	1	1
15.63	mCT	1	1	1	0	1	1	1	1	1	1	1	1
8.17	Vision	1	1	1	1	1	1	1	1	1	1	1	1
8.13	mCT	1	1	0	0	1	1	1	1	1	1	1	1
4.83	Vision	1	1	1	1	1	1	1	1	1	1	1	1
4.93	mCT	1	1	0	0	1	1	1	0	1	1	1	1
2.53	Vision	1	1	1	0	1	1	1	1	1	1	1	1
2.51	mCT	1	0	0	0	1	1	0	0	1	1	1	0
1.37	Vision	1	0	0	0	1	1	1	0	1	1	1	1
1.54	mCT	0	0	0	0	1	0	0	0	1	1	0	0
0.80	Vision	1	0	0	0	1	1	1	0	1	1	1	0
0.79	mCT	0	0	0	0	1	0	0	0	1	1	0	0
0.43	Vision	0	0	0	0	1	1	0	0	1	1	0	0
0.48	mCT	0	0	0	0	0	0	0	0	1	0	0	0
0.26	Vision	0	0	0	0	1	1	0	0	1	1	0	0
0.25	mCT	0	0	0	0	0	0	0	0	0	0	0	0

Supplemental Table S9: Human observer study results for the 9.7-mm sphere.

The human observer study results are separately presented for the examined PET/CT scanners, reconstruction algorithms and emission times. 1 = detected. 0 = undetected.

Sphere Activity Concentration (kBq/ml)	Scanner	OSEM				OSEM+TOF				OSEM+TOF+PSF			
		30 min	16 min	8 min	4 min	30 min	16 min	8 min	4 min	30 min	16 min	8 min	4 min
24.99	Vision	1	1	1	1	1	1	1	1	1	1	1	1
24.85	mCT	1	1	1	1	1	1	1	1	1	1	1	1
15.72	Vision	1	1	1	1	1	1	1	1	1	1	1	1
15.63	mCT	1	1	1	1	1	1	1	1	1	1	1	1
8.17	Vision	1	1	1	1	1	1	1	1	1	1	1	1
8.13	mCT	1	1	1	1	1	1	1	1	1	1	1	1
4.83	Vision	1	1	1	1	1	1	1	1	1	1	1	1
4.93	mCT	1	1	0	0	1	1	1	0	1	1	1	1
2.53	Vision	1	1	1	0	1	1	1	1	1	1	1	1
2.51	mCT	1	0	0	0	1	1	0	0	1	1	1	0
1.37	Vision	1	1	0	0	1	1	1	0	1	1	1	1
1.54	mCT	0	0	0	0	1	0	0	0	1	1	0	0
0.80	Vision	1	1	0	0	1	1	1	0	1	1	1	0
0.79	mCT	0	0	0	0	1	0	0	0	1	1	0	0
0.43	Vision	0	0	0	0	1	1	0	0	1	1	0	0
0.48	mCT	0	0	0	0	0	0	0	0	1	0	0	0
0.26	Vision	0	0	0	0	1	1	0	0	1	1	0	0
0.25	mCT	0	0	0	0	0	0	0	0	0	0	0	0

Supplemental Table S10: SNR for the 3.7-mm sphere.

The SNR values are separately presented for the examined PET/CT scanners, reconstruction algorithms and emission times.

Sphere Activity Concentration (kBq/ml)	Scanner	OSEM				OSEM+TOF				OSEM+TOF+PSF			
		30 min	16 min	8 min	4 min	30 min	16 min	8 min	4 min	30 min	16 min	8 min	4 min
24.99	Vision	4.51	3.35	1.41	1.11	14.55	7.53	4.11	2.78	21.68	11.90	7.07	5.77
24.85	mCT	1.05	1.82	1.04	0.00	3.42	2.50	1.31	0.36	8.05	5.25	3.42	0.81
15.72	Vision	3.51	1.42	0.78	0.31	10.56	8.25	4.48	0.99	12.56	7.81	3.49	1.25
15.63	mCT	1.91	0.59	0.12	0.62	1.47	0.45	0.18	0.41	3.85	2.28	1.02	1.71
8.17	Vision	1.40	1.18	0.99	0.35	1.51	1.15	0.11	0.12	3.18	2.52	0.65	0.08
8.13	mCT	3.03	1.40	0.26	0.47	2.73	2.01	0.17	0.53	2.66	2.98	0.65	1.93
4.83	Vision	1.33	0.07	0.53	0.23	4.15	2.26	1.39	1.30	5.98	2.64	0.92	0.14
4.93	mCT	2.56	2.53	0.42	0.18	1.37	1.40	0.54	0.46	2.44	1.50	0.41	0.56
2.53	Vision	0.46	0.62	0.90	0.49	1.12	0.56	0.62	1.06	1.25	0.37	0.83	1.17
2.51	mCT	0.24	0.57	0.61	2.16	0.49	1.07	1.11	0.26	2.85	2.10	3.09	3.63
1.37	Vision	0.49	0.71	0.60	0.46	0.26	1.09	0.79	0.61	0.84	1.42	0.65	0.74
1.54	mCT	0.32	0.53	0.51	0.42	1.90	0.33	0.36	0.41	0.64	0.60	0.48	0.51
0.80	Vision	0.50	0.03	0.08	1.27	0.25	0.12	0.27	0.09	1.20	0.56	0.79	0.55
0.79	mCT	0.20	0.29	0.08	0.33	0.37	0.46	0.46	0.24	0.60	0.16	0.55	0.25
0.43	Vision	0.51	0.34	0.22	1.79	1.37	5.87	0.26	0.46	2.59	8.32	1.43	0.48
0.48	mCT	1.63	1.49	0.32	0.33	0.66	2.18	0.40	0.25	0.12	1.67	0.32	0.43
0.26	Vision	0.47	0.29	0.45	0.33	2.23	0.03	0.49	0.24	1.53	0.36	0.71	0.42
0.25	mCT	0.34	0.42	0.31	0.20	0.27	0.40	0.27	0.18	0.65	0.64	0.47	0.39

Supplemental Table S11: SNR for the 4.8-mm sphere.

The SNR values are separately presented for the examined PET/CT scanners, reconstruction algorithms and emission times.

Sphere Activity Concentration (kBq/ml)	Scanner	OSEM				OSEM+TOF				OSEM+TOF+PSF			
		30 min	16 min	8 min	4 min	30 min	16 min	8 min	4 min	30 min	16 min	8 min	4 min
24.99	Vision	7.48	8.47	3.97	1.99	15.75	13.13	10.15	6.49	25.92	20.97	15.04	9.95
24.85	mCT	7.85	7.17	3.98	1.56	8.43	7.75	4.59	2.21	16.49	12.51	7.34	3.64
15.72	Vision	6.06	1.65	0.67	0.51	13.93	7.23	7.76	4.90	22.02	12.37	11.06	6.06
15.63	mCT	2.60	1.73	1.67	1.36	6.18	3.15	1.93	1.30	10.09	6.64	4.21	2.30
8.17	Vision	2.73	1.02	0.98	1.46	5.96	5.00	3.78	4.70	11.13	7.94	6.89	8.95
8.13	mCT	2.33	2.49	0.25	0.20	3.66	3.36	0.30	1.23	6.31	6.14	1.70	0.92
4.83	Vision	2.93	2.10	0.62	0.79	6.58	5.81	2.89	0.86	11.02	9.87	5.57	3.43
4.93	mCT	3.63	2.45	0.67	0.08	3.15	4.52	2.12	0.72	3.69	3.48	2.21	0.31
2.53	Vision	0.57	0.14	0.20	0.67	5.71	2.32	0.75	0.27	7.31	4.32	2.73	0.73
2.51	mCT	0.36	0.99	0.23	1.06	1.11	0.09	0.40	0.48	2.44	0.24	0.03	1.68
1.37	Vision	0.04	0.56	0.64	0.54	2.15	0.24	0.59	0.41	4.18	0.47	0.53	0.40
1.54	mCT	0.07	0.18	0.19	0.36	0.01	0.44	0.91	0.27	0.63	0.09	1.83	0.41
0.80	Vision	2.26	1.01	3.77	1.85	3.70	0.25	0.65	0.55	6.81	2.07	1.71	0.71
0.79	mCT	0.48	0.44	0.17	0.31	0.05	0.43	0.27	0.29	0.78	1.84	0.25	0.35
0.43	Vision	0.56	0.58	0.45	0.38	2.38	3.74	0.19	0.38	5.11	6.45	1.77	0.46
0.48	mCT	0.03	0.91	0.44	0.33	0.63	0.06	0.37	0.25	0.11	0.60	0.64	0.42
0.26	Vision	0.84	1.05	0.43	0.42	0.69	0.44	0.46	0.19	1.29	0.37	0.60	0.47
0.25	mCT	0.24	0.32	0.27	0.30	0.32	0.37	0.27	0.18	0.02	0.41	0.43	0.38

Supplemental Table S12: SNR for the 6.5-mm sphere.

The SNR values are separately presented for the examined PET/CT scanners, reconstruction algorithms and emission times.

Sphere Activity Concentration (kBq/ml)	Scanner	OSEM				OSEM+TOF				OSEM+TOF+PSF			
		30 min	16 min	8 min	4 min	30 min	16 min	8 min	4 min	30 min	16 min	8 min	4 min
24.99	Vision	18.50	13.77	9.88	5.09	33.48	24.13	16.91	10.94	64.28	46.36	33.15	21.23
24.85	mCT	12.36	10.82	9.62	4.44	18.34	17.71	13.35	7.49	41.96	33.44	24.34	16.22
15.72	Vision	16.17	8.68	5.72	4.60	26.64	18.96	13.43	7.81	47.61	34.23	25.47	15.33
15.63	mCT	8.79	4.01	3.54	0.69	13.59	8.31	6.09	3.50	32.49	20.73	17.31	12.26
8.17	Vision	10.79	7.13	6.48	4.05	17.20	11.26	7.36	4.53	32.06	20.41	14.54	9.18
8.13	mCT	6.23	4.54	3.02	1.01	9.23	7.18	4.96	3.02	19.83	14.21	8.01	5.22
4.83	Vision	5.76	4.14	1.87	1.14	11.09	8.90	6.89	3.56	19.56	15.23	12.49	6.39
4.93	mCT	6.45	4.75	3.16	2.99	8.32	7.94	5.84	4.13	17.47	15.41	13.66	6.72
2.53	Vision	7.79	5.26	4.68	2.83	8.76	6.40	3.23	3.62	17.39	13.28	7.52	5.75
2.51	mCT	4.34	3.81	0.28	0.89	4.60	2.04	1.86	3.50	12.13	6.69	4.36	6.02
1.37	Vision	1.89	1.25	1.41	1.63	5.84	3.61	1.76	2.01	12.41	7.56	2.74	2.49
1.54	mCT	6.16	4.81	4.51	3.63	7.80	6.15	2.33	0.28	17.36	13.87	4.47	2.04
0.80	Vision	2.34	1.41	0.15	2.45	4.83	5.62	1.93	3.16	8.94	10.29	5.45	4.92
0.79	mCT	1.96	1.20	0.76	1.86	4.50	3.48	6.26	2.19	14.50	6.92	6.50	10.86
0.43	Vision	1.85	1.60	0.79	0.18	3.44	2.31	1.33	2.65	7.06	3.55	2.60	4.67
0.48	mCT	1.31	0.21	0.19	1.49	2.26	1.93	2.36	0.18	5.24	3.86	3.47	1.20
0.26	Vision	0.91	0.37	0.75	0.83	3.61	1.29	0.41	0.37	6.54	2.62	0.05	0.47
0.25	mCT	0.44	0.22	0.31	0.30	0.99	0.25	0.21	0.18	2.06	0.26	0.45	0.14

Supplemental Table S13: SNR for the 7.7-mm sphere.

The SNR values are separately presented for the examined PET/CT scanners, reconstruction algorithms and emission times.

**Sphere Activity Concentration (kBq/ml)**	**Scanner**	**OSEM**				**OSEM+TOF**				**OSEM+TOF+PSF**			
		**30 min**	**16 min**	**8 min**	**4 min**	**30 min**	**16 min**	**8 min**	**4 min**	**30 min**	**16 min**	**8 min**	**4 min**
24.99	Vision	25.43	19.01	12.31	8.64	38.14	26.98	20.44	14.83	73.41	52.04	39.71	28.17
24.85	mCT	14.30	14.00	8.75	4.90	20.57	19.89	15.39	7.61	52.52	42.25	31.57	21.23
15.72	Vision	21.55	15.35	10.40	6.07	32.60	24.73	16.43	9.25	59.13	45.52	30.70	18.73
15.63	mCT	11.90	7.90	5.06	3.26	16.36	11.17	7.59	5.20	42.05	28.19	19.62	13.59
8.17	Vision	15.85	9.78	8.68	6.89	26.90	17.88	13.87	10.07	50.75	34.56	28.15	21.09
8.13	mCT	8.81	6.65	5.41	3.52	12.95	9.53	8.63	9.03	29.63	21.13	21.15	22.27
4.83	Vision	11.20	8.61	4.93	3.69	17.34	11.77	8.25	3.34	30.14	20.29	13.96	9.75
4.93	mCT	8.51	5.28	4.21	1.05	10.34	6.71	3.67	0.74	24.95	16.42	10.53	4.14
2.53	Vision	5.41	2.23	1.51	0.93	9.68	6.96	3.61	2.53	17.39	13.57	7.53	4.69
2.51	mCT	6.10	2.65	1.79	1.98	8.32	4.22	4.77	3.20	20.04	13.07	12.46	9.34
1.37	Vision	4.37	3.65	2.44	2.39	10.34	6.63	4.67	2.25	20.63	14.04	8.79	4.68
1.54	mCT	4.01	2.92	0.65	0.23	5.03	3.32	2.27	2.12	14.54	9.42	4.91	4.49
0.80	Vision	1.54	1.16	0.72	0.42	5.56	4.10	3.86	2.28	10.64	8.85	8.55	6.36
0.79	mCT	4.01	1.46	2.19	0.68	2.38	2.09	1.72	0.25	9.34	8.30	3.99	3.19
0.43	Vision	1.42	1.89	0.78	1.05	5.56	3.49	2.88	4.49	10.00	6.70	6.99	8.38
0.48	mCT	1.86	0.05	0.36	0.08	3.68	1.33	0.91	0.17	9.70	4.83	2.13	1.40
0.26	Vision	1.77	0.81	0.24	0.40	2.03	1.10	0.02	0.06	4.80	2.44	0.82	0.42
0.25	mCT	0.06	0.16	0.24	1.52	0.15	0.01	0.26	0.18	1.17	0.68	1.50	0.37

Supplemental Table S14: SNR for the 8.9-mm sphere.

The SNR values are separately presented for the examined PET/CT scanners, reconstruction algorithms and emission times.

Sphere Activity Concentration (kBq/ml)	Scanner	OSEM				OSEM+TOF				OSEM+TOF+PSF			
		30 min	16 min	8 min	4 min	30 min	16 min	8 min	4 min	30 min	16 min	8 min	4 min
24.99	Vision	30.79	22.99	15.21	8.24	46.98	33.30	24.28	15.21	90.52	64.11	47.26	29.45
24.85	mCT	18.91	19.37	14.27	7.34	22.61	22.06	14.53	7.51	60.25	47.50	32.39	21.05
15.72	Vision	23.50	16.86	11.45	7.59	37.91	28.83	20.59	13.67	68.51	53.23	38.48	27.20
15.63	mCT	14.72	10.11	7.13	4.39	20.09	14.99	10.44	7.73	51.34	38.67	26.72	20.80
8.17	Vision	18.01	12.33	9.34	5.70	27.41	19.46	13.35	8.81	51.32	36.81	26.45	17.66
8.13	mCT	10.97	6.66	4.16	2.30	15.52	10.85	6.90	5.70	40.46	26.66	17.73	12.43
4.83	Vision	13.56	12.00	7.56	5.88	22.08	17.11	12.77	4.81	40.70	30.86	23.52	15.68
4.93	mCT	9.67	8.53	4.97	3.83	11.94	9.83	6.43	3.19	30.22	24.21	16.97	9.54
2.53	Vision	9.94	7.73	5.28	3.25	15.47	11.98	6.63	4.85	28.16	23.03	13.70	8.92
2.51	mCT	8.28	7.06	2.87	0.93	9.05	7.67	4.39	2.26	23.18	17.29	9.83	5.47
1.37	Vision	7.41	4.53	2.65	1.55	12.66	7.89	6.29	2.94	25.10	15.88	13.11	5.64
1.54	mCT	3.19	2.50	2.05	0.36	6.81	4.81	3.89	2.87	17.91	12.72	10.78	6.22
0.80	Vision	4.31	3.45	1.57	0.80	8.35	6.09	3.41	1.77	16.44	13.85	7.84	5.37
0.79	mCT	1.12	1.19	0.02	0.02	3.49	4.11	4.88	0.66	11.50	11.20	5.78	8.10
0.43	Vision	3.45	2.28	1.41	1.28	4.82	3.81	1.93	1.21	9.95	7.34	4.85	1.96
0.48	mCT	1.92	0.73	0.13	0.33	3.08	3.04	1.69	1.29	7.35	9.61	5.25	0.59
0.26	Vision	1.50	1.38	1.49	0.76	3.95	3.13	1.37	1.07	8.78	7.58	2.45	4.01
0.25	mCT	0.37	0.13	3.48	0.30	2.97	3.91	8.43	0.18	6.12	6.95	7.76	2.17

Supplemental Table S15: SNR for the 9.7-mm sphere.

The SNR values are separately presented for the examined PET/CT scanners, reconstruction algorithms and emission times.

Sphere Activity Concentration (kBq/ml)	Scanner	OSEM				OSEM+TOF				OSEM+TOF+PSF			
		30 min	16 min	8 min	4 min	30 min	16 min	8 min	4 min	30 min	16 min	8 min	4 min
24.99	Vision	35.92	27.78	18.72	12.85	52.81	39.00	28.32	20.09	100.62	73.80	54.52	38.37
24.85	mCT	24.68	23.51	16.51	9.46	29.40	27.76	18.37	8.81	76.88	59.12	40.52	25.62
15.72	Vision	30.92	23.23	14.71	8.88	45.98	35.37	23.09	13.55	82.33	64.45	42.74	27.43
15.63	mCT	17.20	12.84	8.14	4.70	21.15	16.44	10.65	7.62	55.17	40.88	26.68	20.00
8.17	Vision	19.23	12.32	9.47	6.59	29.94	20.62	14.88	9.43	55.49	38.19	29.11	18.84
8.13	mCT	15.61	12.56	9.82	6.97	16.52	12.55	9.79	9.07	43.27	31.56	24.75	21.41
4.83	Vision	12.69	10.93	5.62	5.84	20.67	16.03	10.02	6.08	36.80	28.49	19.25	14.19
4.93	mCT	8.62	7.14	3.77	2.68	13.54	11.73	6.75	4.42	34.29	27.84	19.04	10.01
2.53	Vision	10.70	6.88	5.76	4.77	18.91	13.14	9.48	7.03	33.68	24.96	19.21	14.21
2.51	mCT	6.59	3.06	1.74	1.25	9.24	6.78	4.44	1.82	22.64	14.24	10.19	6.90
1.37	Vision	8.75	4.81	2.86	1.60	13.48	8.52	5.31	2.99	25.75	16.48	9.57	4.84
1.54	mCT	4.93	3.86	1.79	2.47	6.10	4.13	3.15	1.87	15.94	10.30	7.29	7.77
0.80	Vision	5.95	5.08	3.03	0.41	9.95	8.56	7.88	2.74	18.43	16.39	15.31	6.28
0.79	mCT	4.19	3.70	1.26	3.66	7.79	5.38	4.02	0.90	22.25	16.09	8.38	4.15
0.43	Vision	2.00	1.43	1.06	1.44	7.02	4.96	4.35	2.84	13.47	9.73	7.95	4.56
0.48	mCT	2.36	1.79	2.92	0.15	3.37	3.48	1.91	0.12	8.45	7.66	5.82	1.70
0.26	Vision	1.15	2.01	1.16	0.40	6.31	4.42	1.96	0.56	14.34	10.51	4.57	2.27
0.25	mCT	0.86	0.70	1.31	1.94	2.21	0.25	0.26	0.18	5.89	1.97	0.04	0.21
